# Detailed analysis of immunologic effects of the cytotoxic T lymphocyte-associated antigen 4-blocking monoclonal antibody tremelimumab in peripheral blood of patients with melanoma

**DOI:** 10.1186/1479-5876-6-22

**Published:** 2008-05-01

**Authors:** Begoña Comin-Anduix, Yohan Lee, Jason Jalil, Alain Algazi, Pilar de la Rocha, Luis H Camacho, Viviana A Bozon, Cecile A Bulanhagui, Elisabeth Seja, Arturo Villanueva, Bradley R Straatsma, Antonio Gualberto, James S Economou, John A Glaspy, Jesus Gomez-Navarro, Antoni Ribas

**Affiliations:** 1 Department of Surgery, Division of Surgical Oncology, University of California Los Angeles (UCLA), Los Angeles, CA, USA; 2 Department of Human Genetics, UCLA, Los Angeles, CA, USA; 3 Oncology Consultants PA, Houston, TX, USA; 4 Pfizer Global Research and Development (PGRD), New London, CT, USA; 5 Department of Medicine, Division of Hematology/Oncology, UCLA; Los Angeles, CA, USA; 6 Department of Ophthalmology, Jules Stein Eye Institute, UCLA Los Angeles, CA, USA; 7 Department of Microbiology, Immunology and Molecular Genetics; 8 Jonsson Comprehensive Cancer Center, UCLA Los Angeles, CA, USA

## Abstract

**Background:**

CTLA4-blocking antibodies induce tumor regression in a subset of patients with melanoma. Analysis of immune parameters in peripheral blood may help define how responses are mediated.

**Methods:**

Peripheral blood from HLA-A*0201-positive patients with advanced melanoma receiving tremelimumab (formerly CP-675,206) at 10 mg/kg monthly was repeatedly sampled during the first 4 cycles. Samples were analyzed by 1) tetramer and ELISPOT assays for reactivity to CMV, EBV, MART1, gp100, and tyrosinase; 2) activation HLA-DR and memory CD45RO markers on CD4^+^/CD8^+ ^cells; and 3) real-time quantitative PCR of mRNA for FoxP3 transcription factor, preferentially expressed by T regulatory cells. The primary endpoint was difference in MART1-specific T cells by tetramer assay. Immunological data were explored for significant trends using clustering analysis.

**Results:**

Three of 12 patients eligible for immune monitoring had tumor regression lasting > 2 years without relapse. There was no significant change in percent of MART1-specific T cells by tetramer assay. Additionally, there was no generalized trend toward postdosing changes in other antigen-specific CD8^+ ^cell populations, FoxP3 transcripts, or overall changes in surface expression of T-cell activation or memory markers. Unsupervised hierarchical clustering based on immune monitoring data segregated patients randomly. However, clustering according to T-cell activation or memory markers separated patients with clinical response and most patients with inflammatory toxicity into a common subgroup.

**Conclusion:**

Administration of CTLA4-blocking antibody tremelimumab to patients with advanced melanoma results in a subset of patients with long-lived tumor responses. T-cell activation and memory markers served as the only readout of the pharmacodynamic effects of this antibody in peripheral blood.

**Clinical trial registration number:**

NCT00086489

## Background

Cytotoxic T lymphocyte-associated antigen 4 (CTLA4) is an activation-induced, type I transmembrane protein of the immunoglobulin superfamily, expressed by recently activated T lymphocytes as a covalent homodimer. It functions as an inhibitory receptor for the costimulatory molecules B7.1 (CD80) and B7.2 (CD86), efficiently competing with the positive costimulatory receptor CD28 [1-5]. Crosslinking of CTLA4 by B7 in the context of T-cell antigen receptor (TCR) engagement inhibits T-cell activation, interleukin (IL)-2 gene transcription, and T-cell proliferation by directly inhibiting TCR signal transduction [3,6].

CTLA4 blockade using the specific antagonistic monoclonal antibodies ipilimumab (formerly known as MDX010 and BMS734016) and tremelimumab (formerly known as CP-675,206 and ticilimumab) reproducibly induce objective tumor responses in a subset of patients with melanoma [7-15]. Despite a wealth of knowledge about the antitumor activity induced by CTLA4 blockade in animal models, the mechanisms that mediate tumor regression in human patients are currently not fully understood [16,17]. Several mechanisms have been postulated: 1) Blocking the negative signaling from CTLA4 expressed on recently activated tumor antigen-specific T cells may boost natural or induced immune responses to cancer cells [3,18]; 2) Anti-CTLA4 antibodies may deplete CD4^+^CD25^+ ^T regulatory cells (Treg) [19], which constitutively express CTLA4 [20], or inhibit reverse signaling to B7 costimulatory molecules expressed by immune suppressive plasmacytoid dendritic cells (pDC) [21-23] or activated T cells [24]; 3) Anti-CTLA4 antibodies may result in the presence of high titers of antibodies against soluble major histocompatibility complex (MHC) class I chain-related protein A (MICA), an immune suppressive MHC class I-like molecule shed by tumor cells [25]; 4) Expression of CTLA4 on T cells increases their motility and interferes with establishment of durable interactions with cells expressing their cognate antigen [26], which may be reverted with monoclonal antibodies; or 5) Anti-CTLA4 antibodies may have direct cytotoxic effects on tumor cells that express CTLA4 [27].

Some of these hypotheses can be studied using modern immune monitoring assays in peripheral blood. Quantification of antigen-specific T-cell responses by MHC tetramer and enzyme-linked immunospot (ELISPOT) assays is often used to assess immune activation in experimental cancer immunotherapy trials [28]. Definition of key methodological parameters (ie, accuracy, precision, and reproducibility) is critical to determine the extent of T-cell expansion that represents a positive immune response. The magnitude of minimum statistically significant changes in the number of circulating antigen-specific T cells compared with baseline levels (defined as the reference change value [RCV]) was recently reported for the tetramer and ELISPOT assays [29]. This calculation provides a robust definition of immune response (either positive or negative) that can be reliably applied to the monitoring of immunomodulatory effects of CTLA4-blocking monoclonal antibodies.

Tremelimumab is a fully human immunoglobulin (Ig)G2 monoclonal antibody with high CTLA4 specificity that antagonizes binding of CTLA4 to B7 costimulatory molecules, resulting in enhanced T-cell activation as demonstrated by increased cytokine production in vitro. Tremelimumab has demonstrated antitumor activity in patients with metastatic melanoma [12]. As with any study using patient-derived samples, the ability to robustly test or rule out a hypothesis is limited by practical constraints of human experimentation [30]. Within these limitations, we set up to test the hypothesis that tremelimumab may alter the number, functional activation or phenotype of immune cells in peripheral blood that may provide information on the mechanism of action of this CTLA4-blocking monoclonal antibody. Therefore, in this report we analyzed immune parameters in the peripheral blood of patients receiving tremelimumab for the treatment of locally advanced or metastatic melanoma with the goal of studying the mechanism of immune activation leading to objective tumor responses.

## Materials and methods

### Study Design and Assessments

Human leukocyte antigen (HLA)-A*0201-positive patients with metastatic melanoma who received tremelimumab monthly at the maximum tolerated dose in a phase I trial [31] were enrolled in an open-label expansion cohort between June 2004 and April 2005, and consented to donate repeated peripheral blood samples while receiving tremelimumab (10 mg/kg monthly) intravenously for up to 24 cycles. This study was conducted according to the Declaration of Helsinki and its amendments and relevant International Conference on Harmonization Good Clinical Practice guidelines. The protocol and consent forms, and all modifications, were approved by the University of California Los Angeles and University of Texas MD Anderson institutional review boards (approval numbers 03-01-059 and IDO3-0090, respectively). All patients provided written informed consent prior to any study procedures. Blood samples (40 mL) were collected from a peripheral vein during the screening period, on day 1 before the first dose of antibody (both baseline samples), on the day of each new dose in subsequent cycles, and 1 and 2 weeks after each monthly dose during the first 4 monthly cycles of therapy. When possible, a final blood sample was collected at the time the patient went off study for whatever reason. All patients underwent baseline and follow-up eye exams every 2 to 4 months throughout study participation following our previously-published eye surveillance protocol [32,33]. The primary endpoint was the determination of immune response to the melanoma-associated antigen recognized by T cells (MART1)-derived epitope MART1_26–36 _by MHC tetramer assay across multiple time points. This was defined as detecting a 99% RCV for this assay and antigen, which corresponds to a minimum 80% change from baseline in the percentage of circulating antigen-specific T cells [29]. Clinical responses were assessed by Response Evaluation Criteria in Solid Tumors (RECIST) [34]. In addition, a clinical benefit response was defined for patients who were felt by the study investigators to have derived unequivocal clinical benefit after tremelimumab despite not meeting the standard criteria for response following RECIST.

### Patient Eligibility

Patients with surgically incurable stage IIIc or IV melanoma were eligible if they met the following major eligibility criteria: HLA-A*0201 positive, baseline level of circulating MART1_26–36_-specific T cells above the lower limit of detection (LLD) by tetramer assay, previously defined as 0.03% of CD8^+ ^T cells [29], disease measurable by RECIST, Eastern Cooperative Oncology Group performance status ≤ 1, and having received at least 2 doses of tremelimumab with samples for immune monitoring collected weekly between both doses. Major exclusion criteria were history of chronic inflammatory or autoimmune disease and presence of active brain metastases.

### Sample Processing, Cryopreservation, and Thawing

Peripheral blood mononuclear cells (PBMC) were isolated by Ficoll-Hypaque (Amersham Pharmacia, Piscataway, NJ) and cryopreserved in liquid nitrogen in Roswell Park Memorial Institute medium (RPMI) (Gibco-BRL, Gaithersburg, MD) supplemented with 20% (all percentages represent v/v) heat-inactivated human AB serum (Omega Scientific, Tarzana, CA) and 10% dimethylsulfoxide (Sigma, St. Louis, MO). Cryopreserved PBMC aliquots were thawed and immediately diluted with RPMI complete media consisting of 10% human AB serum and 1% penicillin, streptomycin, and amphotericin (Omega Scientific). Cells were washed and subjected to enzymatic treatment with DNAse (0.002%, Sigma) for 1 hour at 37°C. Cells were washed again and used immediately or were rested overnight in RPMI complete media in a 6% CO_2 _incubator.

### MHC Tetramer Assay

The following peptide epitopes were used: 1) 2 negative control epitopes: an HLA-A*0201-binding nonrelevant peptide (referred to as Negative peptide from here on) [35], and the HLA-A*0201 immunodominant peptide alpha fetoprotein (AFP)_325–332 _(GLSPNLNRFL) derived from the oncofetal antigen AFP [36]; 2) 2 infectious disease epitopes as positive controls, cytomegalovirus (CMV)pp65_495–503 _(NLVPMVATV) and Epstein-Barr virus (EBV) BMLF1_259–267 _(GLCTLVAML); and 3) 3 HLA-A*0201 immunodominant peptides derived from tumor rejection antigens: MART1_26–35 _(ELAGIGILTV), tyrosinase_368–376 _(YMDGTMSQV) and gp100_209–217 _(ITDQVPFSV). All HLA-A*0201 tetramers were purchased from Beckman Coulter Inc., San Diego, CA, as peptide preloaded reagents, and the assay was performed following the manufacturer's instructions with minor modifications as previously described [29].

### ELISPOT Assays

Interferon gamma (IFN-γ) ELISPOT assays were also performed as previously described [29]. Briefly, PBMC were thawed from different time points and treated with DNAse. HLA-A*0201-transfected K562 (K562/A*0201), provided by Drs. Wolfgang Herr and Cedrik M. Britten (Johannes Gutenberg University, Mainz, Germany), were pulsed with the same peptide epitopes described for the tetramer assay and used as antigen-presenting cells. Then 1 × 10^5 ^PBMC were mixed with 1 × 10^4 ^peptide-pulsed K562/A*0201 in X-Vivo 10 media (BioWhittaker, Walkersville, MD) supplemented with 10% heat-inactivated human AB serum and seeded directly into anti-IFN-γ antibody coated ELISPOT plates for 20 hours.

### Multiplex Cytokine Assay

Cocultures of thawed PBMC and peptide pulsed K562/A*0201 cells were plated as described for ELISPOT assays but were placed in triplicate in flat-bottom 96-well plates. Twenty hours later, supernatants were collected and frozen after centrifugation at 468 g for 10 minutes. Thawed supernatants from different time points were analyzed following the manufacturer's instructions using a 17-plex assay (Bio-Plex Human Cytokine 17-Plex Panel, Bio-Rad Laboratories, Hercules, CA). The cytokines quantified were IL-2, IL-4, IL-6, IL-8, IL-10, granulocyte-macrophage colony-stimulating factor (GM-CSF), IFN-γ, tumor necrosis factor alpha (TNF-α), IL-1β, IL-5, IL-7, IL-12 (p70), IL-13, IL-17, granulocyte colony-stimulating factor (G-CSF), monocyte chemoattractant protein 1 (MCP-1/MCAF), and macrophage inflammatory protein 1 α (MIP-1α). Data were analyzed using Bio-Plex manager software with 5PL curve fitting.

### Multiparameter Surface Flow Cytometry Analysis

Unspecific antibody binding to Fc receptors from thawed PBMC was blocked with 100% adult bovine serum (Omega Scientific). PMBC were then stained using a panel of fluorescein-labeled antibodies against the following T-cell surface antigens: FITC-conjugated-UCHL1 (anti-CD45RO), APC-CY7-conjugated-SK3 (anti-CD4), APC-conjugated-FN50 (anti-CD69) (all from BD Biosciences, San Jose, CA), Pacific Blue-conjugated-S4.1 (anti-CD3) (Invitrogen, Carlsbad, CA), and ECD-conjugated-Immu-357 (anti-HLA-DR) (Beckman Coulter). Cells were fixed with 0.5% paraformaldehyde. Immediately before flow cytometric analysis, 5 μL of 7-amino-actinomycin D (7-AAD) was added to gate out dead cells. The 5 color flow cytometry staining was acquired by a FACSAria using Fluorescence Minus One approach [37]. Analysis was performed with FCS Express (DeNovo Software, Thornhill, Ontario, Canada) software.

### FoxP3 Intracellular Staining

PBMC were first labeled with the following surface antibodies: Pacific Blue-conjugated-S4.1 (anti-CD3), AlexaFluor467-conjugated-RPA-T4 (anti-CD4), APC-CY7-conjugated-M-A251 (anti-CD25). Intracellular staining for FoxP3 protein was performed following the manufacturer's instructions using the PE-conjugated-PCH101 anti-FoxP3 antibody (eBioscience, San Diego, CA). Flow cytometric analysis was performed as described above.

### Real-Time Quantitative Polymerase Chain Reaction (QPCR) for FoxP3

Total RNA was extracted from thawed PBMC using RNeasy mini kit (Qiagen, Valencia, CA). Human FoxP3 mRNA expression was quantified using the iScript One-Step Quantitative reverse-transcriptase PCR kit (Bio-Rad) in an Opticon 2 (MJ Research, Ramsey, MN), and sample concentrations were corrected with human 18S rRNA [38]. Amplification was conducted in a total volume of 25 μL for 40 cycles of 15 seconds at 95°C, 30 seconds at 60°C and 30 seconds at 72°C, with 2 beginning steps: 10 minutes at 50°C (to convert RNA to cDNA) and 15 seconds at 95°C (to inactivate reverse transcriptase). Samples were run in triplicate. FoxP3 primers were forward 5'-CAA GTT CCA CAA CAT GCG AC-3'; and reverse, 5'-ATT GAC TGT CCG CTG CTT CT-3' [39]. 18S rRNA, primers were forward 5'-GC-CGA-AGC-GTT-TAC-TTT-GA-3' and reverse 5'-TCC-ATT-ATT-CCT-AGC-TGC-GGT-ATC-3' [38].

### Tumor Processing for Tetramer Assay

To generate a single-cell suspension and analysis of tumor infiltrating lymphocytes (TIL), tumors obtained from an outpatient excisional biopsy were decapsulated, minced with sterile surgical blades and enzymatically digested for 1 to 2 hours with DNAse I (0.1 mg/mL, Sigma) and collagenase D (1 mg/mL, Boehringer Mannheim, Indianapolis, IN) in 100 mL of AIM-V^® ^media (Gibco-BRL). Cells were plated in flasks in RPMI culture media and allowed to adhere for 2 hours. At that time, nonadherent cells, enriched for TIL, were collected and cryopreserved for tetramer analysis.

### Statistical Analysis

The previously defined 99% RCV [29] was applied to detect statistically significant changes in values above the lower limit of detection for the tetramer and ELISPOT assays. Data from all assays were normalized to an arbitrary scale from 0 to -4 or +4 change from baseline for the generation of heat maps for unsupervised and supervised hierarchical clustering of results. The mean of the results of 2 predosing samples was given a value of 0, and postdosing changes could be either positive or negative. For tetramer and ELISPOT assay results, positive changes < 99% RCV were scored as 1, twice the RCV was scored as 2, 3 times the RCV as 3, and 4 was scored for any result beyond this point. Negative changes compared with baseline were scored with the corresponding negative values from -1 to -4. The percentage change from baseline for surface flow cytometry results and QPCR was calculated for FoxP3 mRNA as described by Maker et al [40]. A value of 1 was assigned for a positive or negative percentage change between 0 and 50% from baseline, 2 for percentage changes between 51% and 100%, 3 for percentage changes between 101% and 150%, and 4 for changes beyond 151%. Samples were analyzed by pairwise average-linkage cluster analysis [41] using dChip software initially developed for genome-wide microarray expression data analysis. In this analysis, data vectors typically assigned as gene probe sets were substituted with the aforementioned transformed tetramer, ELISPOT, flow cytometry, and QPCR serial data points. Samples were assigned vector columns, and assay points were assigned vector rows. In addition, a paired *t *test was used to determine significant changes in pre- and postdosing levels of the surface markers HLA-DR and CD45RO.

## Results and Discussion

### Patient Characteristics

Twenty-three HLA-A*0201-positive patients with surgically incurable stage IIIc or IV MART1-positive melanoma provided a baseline blood sample for screening analysis of circulating CD8^+ ^T cells specific for MART1_26–35 _epitope. Patients with a baseline value within the measurable range for the tetramer analysis (≥ 0.03%) were selected. The inclusion of this criterion was based on the goal of quantification of both positive and negative changes in the number of circulating MART1_26–35_-specific T cells after treatment with tremelimumab. Patients with baseline levels of MART1_26–35_-specific T cells below the assay LLD would not contribute to assessing a potential decrease in circulating T cells specific for this antigen upon treatment with CTLA4 blocking antibodies [29], a possibility that has not been excluded in prior studies [8,9,14,19,40,42]. Fifteen patients were enrolled and 12 received 2 or more monthly doses of tremelimumab; 2 patients experienced rapid tumor progression before the second scheduled dose, and 1 patient never received the first dose due to rapid deterioration of performance status. The 12 patients who received at least 2 doses of tremelimumab were considered evaluable for immune-monitoring assays, and their characteristics are summarized in Table 1.

**Table 1:** Patient characteristics, toxicities, and response to therapy

**Patient code**	**Age**	**Sex**	**Stage**	**Disease sites**	**Prior therapy**	**Tremelimumab doses, no.**	**Treatment-related toxicity, G2/G3**	**Best response**
1103	80	M	IVc	Liver		2	Diarrhea G2	PD
				Lung				
1104	63	M	IVb	LN	Temozolomide, thalidomide, IL-2	2		PD
				Lung				
				Liver				
				Adrenal				
				Kidney				
1105A	61	M	IVc	LN	Cisplatin, vinblastine, temozolomide, IL-2, IFN, lenalidomide, MART1/DC	11	Hepatitis G3 Acne Rosacea G2	SD × 11 mos
				Lung				
				Stomach				
1105P	55	F	IVc	LN	Cisplatin, vinblastine, dacarbazine, paclitaxel, gefitinib, BCG, IL-2, IFN	4		PD
				Lung				
				Liver				
1106	52	F	IVc	LN	Cisplatin, vinblastine, dacarbazine	4	Diarrhea G3 Rash G2	PD
				Lung				
				Liver				
1107	57	M	IVc	LN	Heat shock	2		PD
				Liver	Protein			
1108	55	F	IIIc	SC	GM-CSF	5	Hepatitis G3 Hypophysitis G2	CR × 39+ mo.
				LN				
1109	54	M	IVa	LN	ILX 651	3	Leuko-cytoclastic vasculitis G3	PD
1110	34	M	IVa	SC	IL-2	3		PD
				LN				
1111	90	M	IIIc	Skin	BCG,	8	Diarrhea G2	PR × 34+ mo.
				LN	AMG 706			
1113	63	M	IVa	LN	IFN	22	Hypophysitis G2 Elevated trans-aminases G2 Asthenia G2 Arthralgia G2 Grover's G2 Uveitis G2	pPR × 33+ mo.
1114	61	M	IVa	LN	IFN, temozolomide	2	Colitis G3	SD × 8 mo.

No. = Number; G = Grade; M = Male; PD = Progressive disease; LN = Lymph node; IL-2 = Interleukin-2; SD = Stable disease; mo.= Months; IFN = Interferon; MART1 = Melanoma-associated antigen recognized by T cells; DC = Dendritic cells; F = Female; BCG = Bacillus Calmette-Guérin; SC = Subcutaneous; GM-CSF = Granulocyte-macrophage colony-stimulating factor; CR = Complete response; PR = Partial response; pPR = Pathological partial response.

### Toxicities

**Figure 1:**
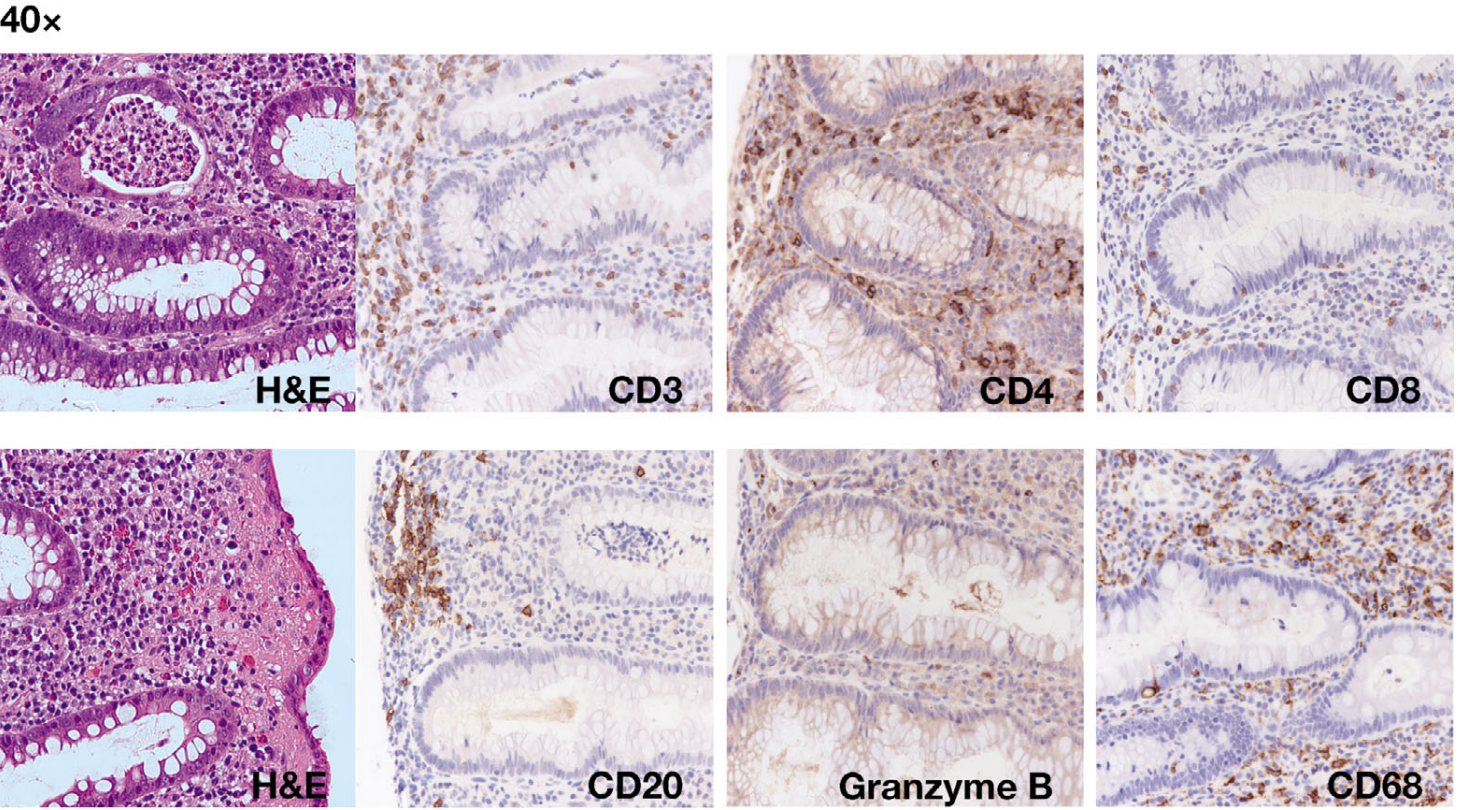
Grade 3 colitis with tremelimumab. High magnification images of a biopsy of the colon in patient 1114. Sections from paraffin-embedded tissue were stained by hematoxylin-eosin (H&E) and CD3, CD4, CD8, CD68, and granzyme B by immunohistochemistry using commercially available antibodies.

None of these patients developed a grade 4 treatment-related toxicity; however, 5 patients experienced treatment-related grade 3 toxicities. Two (1105A and 1108) developed marked increase in transaminases after 11 and 5 doses, consistent with hepatitis (Table 1). Acute viral hepatitis was ruled out in both cases. In both cases this was a reason for study discontinuation. Although neither patient received corticosteroids, both fully recovered within 2 months. One of these patients underwent 5 daily therapeutic plasma exchange procedures with efficient clearance of detectable levels of circulating tremelimumab. Two patients (1106 and 1114) developed grade 3 diarrhea after 4 and 2 doses. One underwent colonoscopy and biopsy, which demonstrated histologically proven colitis with lymphomonocytic infiltrates predominantly by CD4^+ ^T helper cells and CD68^+ ^macrophages (Figure 1). A final patient (1109) developed a generalized rash, which was described as leukocytoclastic vasculitis at biopsy. Clinically significant grade 2 toxicities included 2 cases of panhypopituitarism suggestive of hypophysitis, patient 1108 after 5 doses and patient 1113 after 22 doses of tremelimumab. This later patient also developed anterior uveitis after 21 doses that rapidly improved following treatment with corticosteroid-containing eye drops.

### Clinical Responses

**Figure 2:**
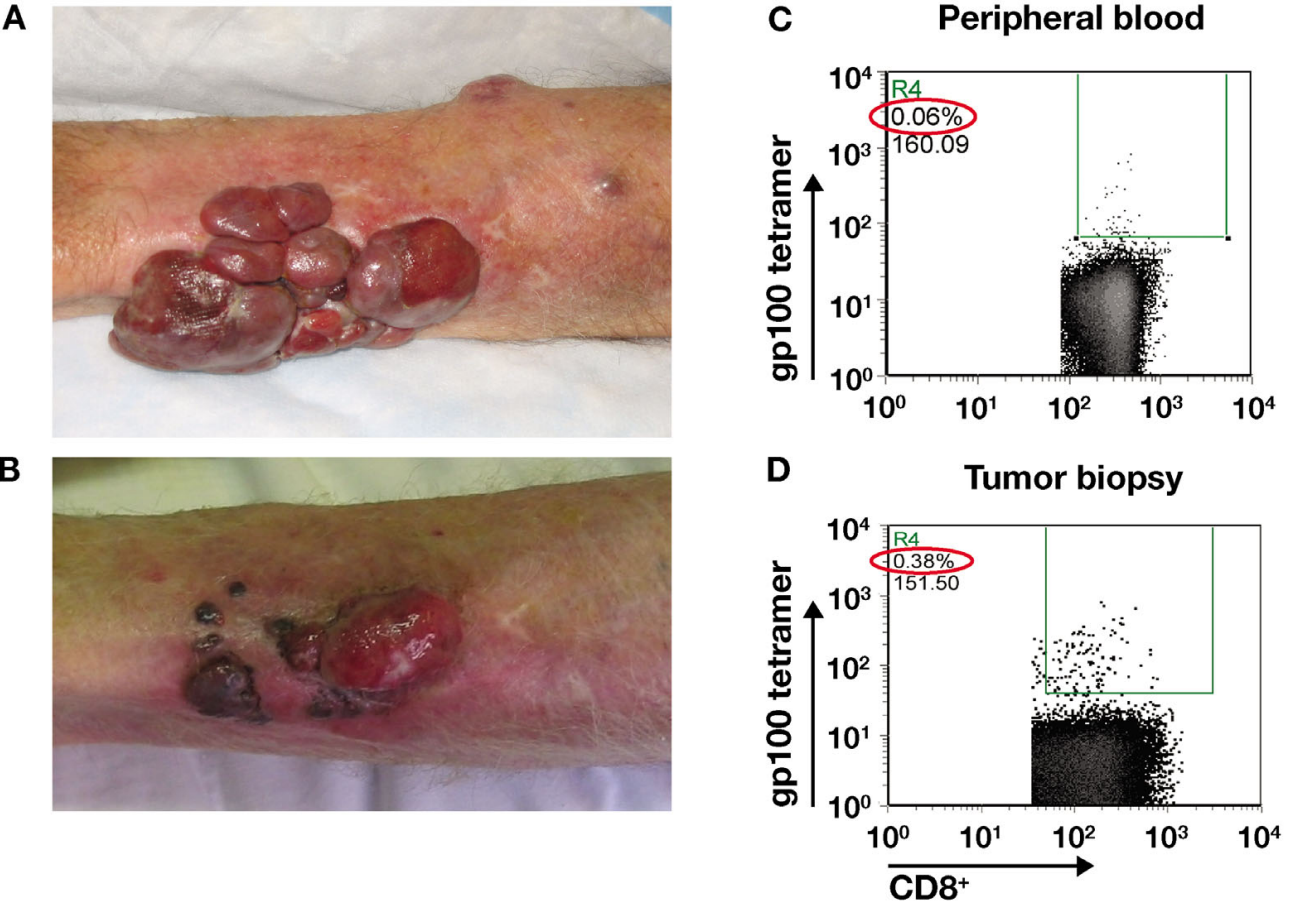
Clinical response to tremelimumab. (A) Baseline picture of patient 1111 intransit metastases, which were progressing after experimental therapy with an antiangiogenic agent. (B) Follow-up picture 5 months after initial dosing while receiving monthly tremelimumab at 10 mg/kg. (C) Dot plot of MHC tetramer analysis taken from peripheral blood at cycle 3. (D) Dot plot of MHC tetramer analysis of tumor infiltrating lymphocytes from a tumor biopsy of a regressing melanoma lesion at cycle 3. MHC = Major histocompatibility complex.

One patient (1108) achieved a complete response of 2 nodal metastases that were proven to be melanoma at baseline by fine needle aspiration. This response is ongoing 39+ months later. One patient (1111) had a major partial response of bulky in-transit metastasis, and remains relapse-free at 34+ months (Figure 2A and 2B). A third patient (1113) did not qualify as a responder by RECIST criteria but was determined to have unequivocal evidence of clinical benefit. A [^18^F]fluorodeoxy-glucose (FDG) positron-emission tomography (PET)-positive 4-cm psoas muscle mass became PET negative after 9 doses. Upon surgical resection, pathological analysis showed a 90% regression of melanoma with no disease progression at 33+ months. This patient is listed as having a pathological partial response. Only 1 of 5 patients with grade 3 toxicity had an objective clinical response.

### MHC Tetramer Assay

**Figure 3:**
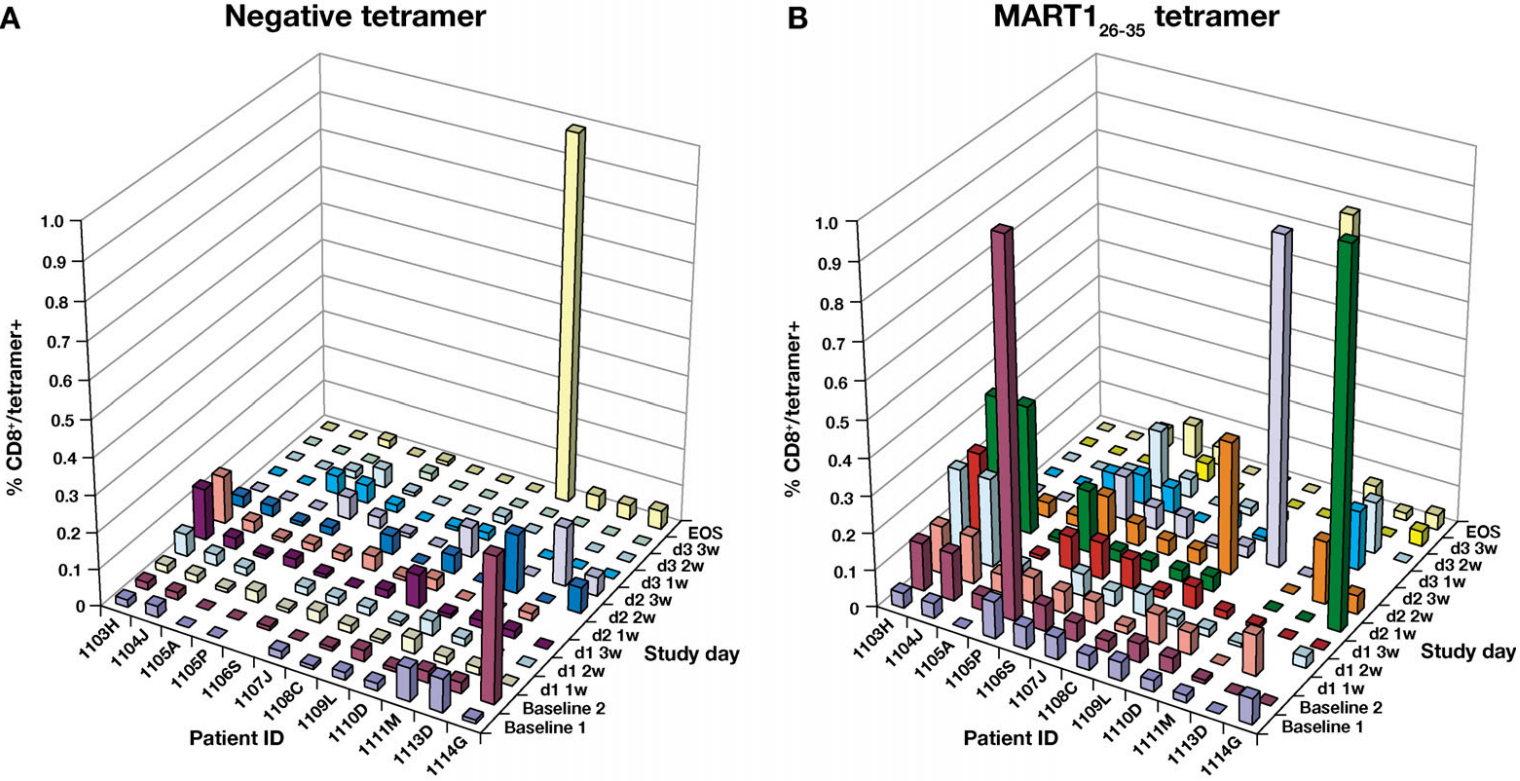
Major histocompatibility complex tetramer analysis in patients receiving tremelimumab. Bar graphs represent percentages of CD8^+ ^T cells specific for (A) negative control epitope and (B) MART1_26–35 _in samples from all patients and available time points in the tetramer analyses. MART1 = Melanoma-associated antigen recognized by T cells; EOS = End of study.

T cells from treated patients were screened to determine whether responses to tumor-specific antigens developed as a result of treatment with tremelimumab. A mean of 2 baseline and 7 follow-up (range, 5 to 9) blood draws per patient were tested. Samples were analyzed by flow cytometry for negative tetramer-specific MART1_26–35_-specific CD8^+ ^T cells. Percentages of negative tetramer-specific and MART1_26–35_-specific CD8^+ ^T cells at each time point indicated increases beyond the 99% RCV in 4 patients on more than 1 occasion (Figure 3). However, these were spikes of peripheral appearance of melanoma antigen-specific T cells at isolated time points, as opposed to persistent elevations in several blood draws taken after dosing. Patient 1111, who had an objective clinical response to therapy, underwent biopsy of a responding lesion. MHC tetramer analysis of nonadherent cells from this lesion demonstrated a 6-fold enrichment of gp100_209–217_-specific CD8^+ ^T cells among total CD8^+ ^T cells compared with peripheral blood (Figure 2C and 2D).

**Figure 4:**
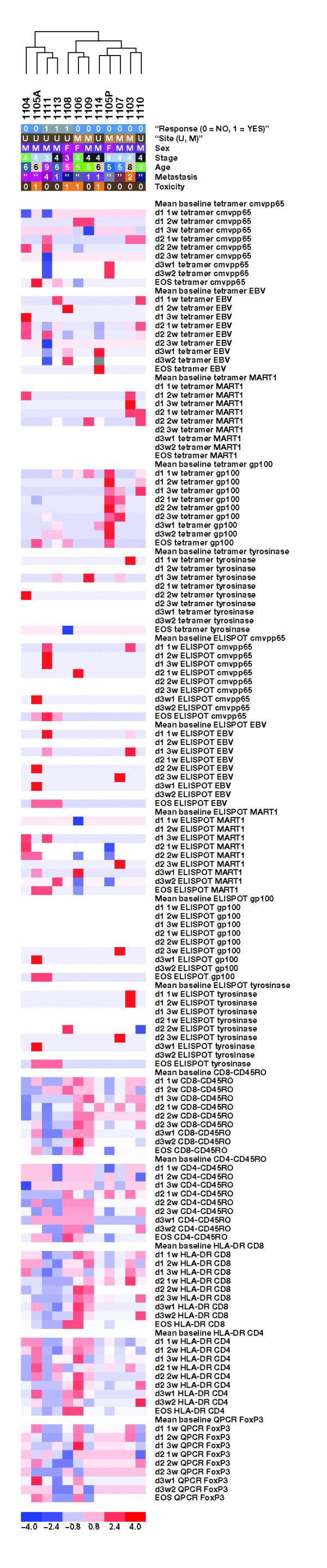
Unsupervised hierarchical clustering of clinical parameters and tetramer, ELISPOT, HLA-DR, CD45RO, and FoxP3 QPCR for all patients and available time points. Each column represents results for 1 patient. The first 5 rows describe clinical features of patients, including response to therapy (0 = No response; 1 = Response), site (U = University of California, Los Angeles; M = MD Anderson), sex (M = Male; F = Female), stage (3 = IIIc; 4 = IV), age, and metastasis (0 = Lung; 1 = Nodes; 2 = Liver; 3 = Subcutaneous; 4 = Skin; 5 = Stomach; 6 = Adrenal; 7 = Kidney). The rest of the rows represent test results at different time points after dosing. White represents no change (0 value) or absence of data (which is not included in the clustering algorithm). Red and shades of red represent positive changes on a scale of 1 to 4. Blue and shades of blue represent negative changes on a scale of 1 to 4. Data are normalized to a -4 to +4 scale as described in Table 2. ELISPOT = Enzyme-linked immunospot; HLA = Human leukocyte antigen; QPCR = Quantitative polymerase chain reaction; EBV = Epstein-Barr virus; MART1 = Melanoma-associated antigen recognized by T cells; EOS = End of study.

To efficiently present all of the data describing positive or negative changes across 12 patients, multiple time points, and the different test epitopes (a total of 719 assay results), the data were converted to a -4 to +4 scale based on the 99% RCV (as described in the Materials and Methods and Table 2) and color coded in shades of blue for negative changes and red for positive changes. These data were compiled in a heat map similar to the ones used to analyze data from gene expression profiling (Figure 4). As described for MART1_26–35_-specific T cells, no definite trend was observed for changes over time in the proportion of CD8^+ ^T cells reacting to any melanoma-(gp100_209–217_, tyrosinase_368–376_) or infectious disease-(EBV BMLF1_259–267_, CMVpp65_495–503_) specific antigen in any patient.

**Table 2:** Percent change from mean baseline normalized to a scale of ± 4

**Assay**	**Epitope**	**1**	**2**	**3**	**4**
MHC tetramer	EBV BMLF1_259–267_	≥85.89	≥171.78	≥257.67	≥343.56
	CMVpp65_495–503_	≥69.89	≥138.58	≥207.87	≥277.16
	MART1_26–35_	≥107.29	≥214.58	≥321.87	≥429.16
	Tyrosinase_368–376_	≥107.29	≥214.58	≥321.87	≥429.16
	gp100_209–217_	≥107.29	≥214.58	≥321.87	≥429.16

IFN-γ ELISPOT	EBV BMLF1_259–267_	≥246.85	≥493.7	≥740.55	≥987.4
	CMVpp65_495–503_	≥192.4	≥384.8	≥577.2	≥769.6
	MART1_26–35_	≥264.1	≥528.2	≥792.3	≥1,056.4
	Tyrosinase_368–376_	≥264.1	≥528.2	≥792.3	≥1,056.4
	gp100_209–217_	≥264.1	≥528.2	≥792.3	≥1,056.4

HLA-DR		0 – 50	51 – 100	101 – 150	150 – 200+

CD45RO		0 – 50	51 – 100	101 – 150	150 – 200+

QPCR		0 – 50	51 – 100	101 – 150	150 – 200+

MHC = Major histocompatibility complex; EBV = Epstein-Barr virus; CMV = Cytomegalovirus; MART1 = Melanoma-associated antigen recognized by T cells; IFN-γ = Interferon gamma; ELISPOT = Enzyme-linked immunospot; HLA = Human leukocyte antigen; QPCR = Quantitative polymerase chain reaction.

### IFN-γ ELISPOT Assay

The heat map in Figure 4 also presents all the data generated by ELISPOT assay enumerating cells producing IFN-γ after short ex vivo peptide stimulation. Results are color coded following the -4 to +4 scale range with negative and positive changes defined by the 99% RCV compared with the results from the 2 baseline samples (Table 2). The data summarize replicate results from a mean of 9 (range, 6 to 11) time points analyzed from the 12 study patients testing reactivity to the negative control and test antigens for a total of 1,694 sample results. As with the tetramer assay, there was no evidence of a consistent change over time in the assay results for any of the tested epitopes.

### Multiplex Cytokine Production Upon Antigen Exposure

The possibility that IFN-γ may not be the most significant cytokine to define the functional status of antigen-specific T cells after CTLA4 blockade was explored. Therefore, we set up cocultures of 2 × 10^5 ^PBMC and 2 × 10^4 ^K562/A*0201 cells pulsed with either negative, MART1_26–35_, tyrosinase_368–376, _and gp100_209–217 _peptides in parallel with the ELISPOT assays of patients 1108, a responder, and 1110, a nonresponder. Twenty hours later supernatants were collected and analyzed with a bead-based multiplex assay of 17 cytokines (background [PBMC plus K562/A*0201] was subtracted from all samples). The PBMC cytokine profile from patient 1110 was under the assay limit of detection except for baseline IL-8, MCP-1, and MIP-1β. In contrast, cell supernatant from patient 1108 showed that IL-6, IL-8, G-CSF, MCP-1, and MIP-1β increased after dose 1 (week 2) and dose 2 (week 3) only. IFN-γ, TNF-α, IL-7, and IL-13 demonstrated a small increase after dose 2 (week 3). The cytokines IL-2, IL-4, IL-5, IL-10, IL-17, GM-CSF, and IL-12(p70) were not detected in supernatants of this patient (data not shown). From this preliminary analysis, we concluded that multicytokine analysis in these 2 representative patients was unlikely to provide additional information beyond the information obtained by the standardized IFN-γ ELISPOT.

### Analysis of T-Cell Activation and Memory Markers

Based on the emerging results suggesting that there was no consistent change in the number of circulating CD8^+ ^T cells specific for melanoma or infectious disease antigens analyzed by either biochemical (tetramer) or functional (ELISPOT and multicytokine) assays, we sought evidence of T-cell activation regardless of antigen specificity. Cell surface expression of the activation-induced T-cell marker HLA-DR (MHC class II molecule) and the T-cell memory marker CD45RO have been reported to be increased after administering CTLA4-blocking monoclonal antibodies [8,9,14,19,40]. Ten patients had at least 1 baseline and 1 postdosing aliquot of PBMC available for this analysis, for a total of 81 analyzed samples. There was no overall significant increase in the expression of these 2 surface markers on either CD4^+ ^and CD8^+ ^T-cell subsets comparing pre- and postdosing samples, although HLA-DR and CD45RO were significantly increased on CD4^+ ^cells after the first dose (paired *t *test *P *= .02 and .01, respectively), and CD45RO was significantly increased on CD8^+ ^cells after cycles 1 and 2 (paired *t *test *P *= .009 and .04, respectively). Results were then converted to percentage change from baseline as described by Maker et al [40] and assigned a score from -4 to +4 (Table 2) to allow compiling of data in the heat map (Figure 4).

### Analysis of FoxP3 mRNA and Intracellular Protein

An alternative possibility was that CTLA4-blocking antibodies affected CTLA4-positive Treg. Other groups have explored this possibility with divergent results [14,19,40]. In 8 patients (including the 3 patients with clinical benefit), aliquots of at least 1 baseline and 1 follow-up PBMC sample were available for analysis using QPCR to quantitate FoxP3 mRNA transcripts. We failed to detect a statistically significant change (either positive or negative) between pre- and postdosing results. Results from the 56 total tested samples were converted to the same scale used for the flow cytometry data (Table 2) and compiled in the heat map (Figure 4).

There were not enough cells derived from the 40 mL peripheral blood draws to perform functional assays of Treg-mediated suppression. In a further attempt to test a possible effect of tremelimumab on circulating Treg, expression of the intracellular FoxP3 protein in cells with the CD3^+^CD4^+^CD25^high ^phenotype was analyzed by flow cytometry. This included at least 1 baseline and 1 postdosing representative sample taken from 2 responders (1108 and 1111) and 3 nonresponders (1103, 1105A, and 1110), for a total of 19 analyzed samples. Data were again converted to percent change from baseline, scored with values between -4 and +4, and compared with results from FoxP3 mRNA analysis at the same time points. There was good concordance between the results of both assays at 9 time points, whereas at the other 10 time points both assays diverged at values ≥ 2 or ≤ -2 (data not shown). These discrepancies underscore the difficulty in efficiently quantifying Treg using currently available approaches.

### Unsupervised Hierarchical Relations Between Immune Monitoring and Clinical Results

**Figure 5:**
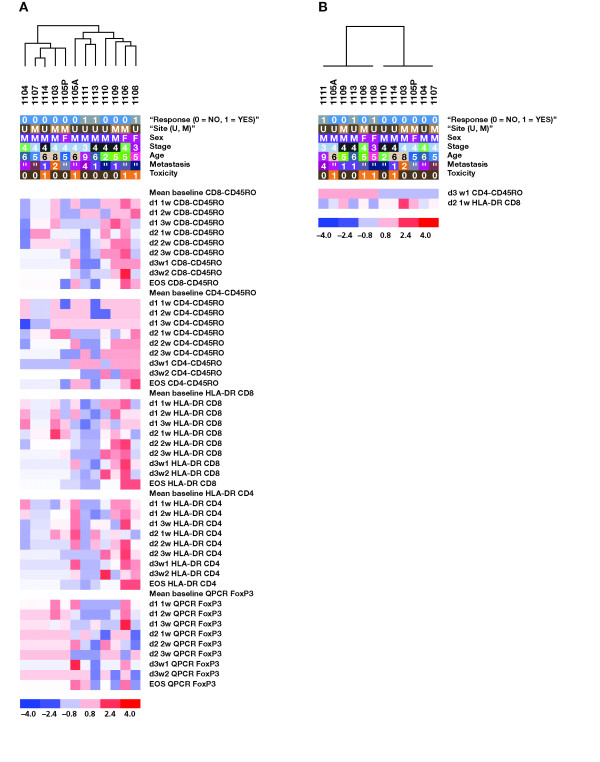
Clustering of clinical parameters and HLA-DR and CD45RO surface staining. (A) Unsupervised clustering of the results from surface staining of CD4^+ ^and CD8^+ ^cells for the T-cell activation marker HLA-DR and the T-cell memory marker CD45RO. (B) Supervised clustering of immune response parameters according to response to therapy. Each column represents results for 1 patient. The first 5 rows describe clinical features of patients, including response to therapy (0 = No response; 1 = Response), site (U = University of California, Los Angeles; M = MD Anderson), sex (M = Male; F = Female), stage (3 = IIIc; 4 = IV), age, and metastasis (0 = Lung; 1 = Nodes; 2 = Liver; 3 = Subcutaneous; 4 = Skin; 5 = Stomach; 6 = Adrenal; 7 = Kidney). The rest of the rows represent test results at different time points after dosing. White represents no change (0 value) or absence of data (which is not included in the clustering algorithm). Red and shades of red represent positive changes on a scale of 1 to 4. Blue and shades of blue represent negative changes on a scale of 1 to 4. Data are normalized to a -4 to +4 scale as described in Table 2. All test results were included in the analysis, and the graph represents the results that best discriminate clinical responders (1108, 1111, 1113) from nonresponders, setting a 1.2-fold difference as the cutoff. Each column and row is presented as described in Figure 4, and the data are normalized to a -4 to +4 scale as described in Table 2. HLA = Human leukocyte antigen; EOS = End of study; QPCR = Quantitative polymerase chain reaction.

The data compiled in the heat map presented in Figure 4 contain all available results of postdosing values compared with the mean of the 2 baseline assay results (with a score of 0). Because the data were converted to a normalized scale of -4 to +4 based on what were determined to be biologically significant changes over baseline, a hierarchical unsupervised clustering analysis could be performed to process all these data in a collective but exploratory approach. This analysis segregated the study patients randomly, with no clustering of patients by clinical response (Figure 4). Unsupervised hierarchical clustering also did not separate patients with grade 2/3 treatment-related toxicities from patients without treatment-related toxicity. We then conducted more limited unsupervised clustering including the results of the tetramer, IFN-γ ELISPOT, or FoxP3 QPCR data alone. Again, patients clustered randomly. However, when we conducted the unsupervised clustering for HLA-DR and CD45RO markers, the 3 clinical responders segregated along with several nonresponders with evidence of inflammatory toxicity (1105A with grade 3 hepatitis, 1106 with grade 3 diarrhea, 1109 with grade 3 leukocytoclastic vasculitis). This segregation was statistically significant (*P *= .05; Figure 5A) and suggests that patients with an objective tumor response and/or toxicity after administration of tremelimumab have a more active immune response detectable in peripheral blood.

### Supervised Hierarchical Relation of Immunologic Monitoring Data According to Clinical Responses

The data set was then examined to explore which assay results better segregated the 3 clinical responders from nonresponders (Figure 5B). Increase in CD45RO on CD4^+ ^cells after 3 doses and increase in HLA-DR on CD8^+ ^cells after 2 doses significantly differentiated responders from nonresponders (2-fold cutoff difference, *P *= .03). These data strengthen the correlation between nonantigen-specific markers of immune activation on T cells and antitumor activity.

## Conclusion

Several reports have described immune monitoring in peripheral blood of patients receiving CTLA4-blocking monoclonal antibodies [8,9,14,19,40,42]. These prior studies did not collect repeated samples allowing time-course analysis of circulating antigen-specific T cells for melanoma and infectious-disease antigens. Analysis of a single time point may miss transient changes (either positive or negative) in circulating cells, or may result in biased results given our prior observation of occasional peaks of circulating T cells at isolated time points [42]. Therefore, we decided to collect a time course of blood samples from consenting patients treated with a fixed dose and schedule of the CTLA4-blocking antibody tremelimumab.

We selected a cohort of HLA-A*0201-positive patients with MART1-positive melanoma who had received at least 2 doses of tremelimumab and had a measurable level of circulating MART1_26–35_-specific T cells to allow analysis of the primary study endpoint. We acknowledge that this is a selected population of patients, and this analysis was aimed at testing both positive and negative changes in antigen-specific T cells in peripheral blood. Contrary to what has been reported by others [9,14], we did not observe a significant correlation between the development of toxicities that may have an autoimmune mechanism of action with clinical responses. A limitation of our analysis is the small number of patients, resulting in lower power to detect statistically significant correlations. However, we did note that certain toxicities, like hepatitis and anterior uveitis, may be more common with repeated dosing, something that has been previously described for hypophysitis [43]. Two unique skin toxicities with tremelimumab were noted in our patients, leukocytoclastic vasculitis and Grover's disease of the skin.

Our data confirm observations by others [8,9,19,40] that administration of CTLA4-blocking antibodies to patients with melanoma does not result in the sustained expansion of circulating melanoma antigen-specific CD8^+ ^T cells. It further rules out that this intervention decreases the number of circulating melanoma antigen-specific T cells. However, the observed marked enrichment of gp100_209–217_-specific CD8^+ ^T cells in regressing tumor tissue compared with peripheral blood in 1 patient suggests that sampling of circulating antigen-specific T cells might miss overall melanoma antigen-specific T-cell expansion in the tumor. This may be particularly relevant for studies without time-course sampling. The occasional peaks of increased numbers of melanoma-circulating antigen-specific T cells detected in several patients may reflect peaks of T-cell expansion at different time points that may not persist because the cells accumulate in tumors. We reasoned that this possibility could be better analyzed when the data were studied in an unbiased manner using unsupervised clustering analysis of data presented in heat maps. However, even with this analysis, we were unable to correlate the activation or expansion of circulating melanoma antigen-specific CD8^+ ^T cells with clinical benefit to CTLA4 blockade. It is certainly possible that the 3 melanoma antigens studied may not be relevant for CTLA4 blockade-induced melanoma regressions. However, these 3 antigen epitopes have been repeatedly recognized as HLA-A*0201 immunodominant peptides in patients with immunotherapy-induced tumor regressions [44].

The constitutive expression of CTLA4 on Treg has raised the possibility that administration of anti-CTLA4 antibodies may deplete or modulate the function of these professional immune suppressive cells [45,46]. However, neither ipilimumab nor tremelimumab were selected as depleting antibodies for CTLA4-positive cells. In fact, both antibodies were selected as blocking antibodies to CTLA4 intended to activate cells expressing their target but not kill them. Furthermore, tremelimumab is an IgG2 antibody, an immunoglobulin subtype unlikely to fix complement or induce antibody-mediated cellular cytotoxicity [47]. Treg are difficult to study at the cellular level because they have a surface phenotype that is indistinguishable from chronically activated T helper cells. They can be detected by the intracellular expression of the Treg-specific transcription factor FoxP3 [48,49]. FoxP3 mRNA transcripts in a population of PBMC can be quantitated by QPCR; however, this technique does not allow efficient enumeration of Treg at the cellular level. Intracellular FoxP3 protein staining can be achieved by multicolor flow cytometry after cell permeabilization, but it is currently unclear how these 2 techniques compare and the bias of each analytical methodology. The gold standard assay for detecting Treg is the determination of their functional ability to inhibit the proliferation of clonally expanded T cells [20]. However, insufficient quantities of blood were collected to perform this type of analysis. Therefore, our findings of no evidence of Treg depletion in peripheral blood samples by FoxP3 QPCR and intracellular staining should be interpreted with caution. Paired FoxP3 mRNA quantitation and Treg functional analysis have been reported by Maker et al [40]. These investigators concluded that administration of the CTLA4-blocking antibody ipilimumab to patients with melanoma neither depleted Treg nor downregulated their functional immune suppressive activity. Our data and the data reported by Maker et al are in contrast to data presented by Reuben et al [14] who suggested that Treg defined by flow cytometry may be preferentially depleted in patients with an objective response to the CTLA4 blocking antibody tremelimumab, and O'Mahony et al [19] who suggested that Treg defined by FoxP3 QPCR may transiently decrease in peripheral circulation 3 days after dosing and recover 4 weeks later.

We analyzed tumor and infectious disease antigen-specific CD8^+ ^T-cell immune monitoring, FoxP3 mRNA quantitation, and T-cell activation/memory marker surface staining concurrently to detect trends relating to clinical parameters. We considered applying a stepwise regression analysis to search for variables that are significantly associated with clinical response. However, our sample size was too small for such an analysis, especially considering that this cohort included only 3 clinical responders. Therefore, to accomplish the goal of generating an overall evaluation of immune monitoring data, we turned to cluster analysis as a means to look at the data in an exploratory fashion. Markers of T-cell activation (HLA-DR) and memory phenotype (CD45RO) after dosing with tremelimumab segregated the 3 patients with clinical benefit together with 4 patients with inflammatory toxicity. Therefore, we conclude that tremelimumab does not increase the number or function of antigen-specific CD8^+ ^T cells in peripheral blood nor decrease FoxP3 transcripts but appears to generally enhance T-cell activation and differentiation. Detection of T-cell activation and memory markers may be useful as readout of this antibody's ability to activate the immune system, which may lead to pharmacodynamic effects consistent with its observed antitumor activity. Sampling of T cells in tumors may provide additional information, and future studies should include immune monitoring in tumor tissues.

## Competing interests

The following authors have no competing interests to report: Begoña Comin-Anduix, Yohan Lee, Jason Jalil, Alain Algazi, Pilar de la Rocha, Elisabeth Seja, Arturo Villanueva, Bradley R. Straatsma, James S. Economou, and John A. Glaspy. Viviana A. Bozon, Cecile A. Bulanhagui, Antonio Gualberto, and Jesus Gomez-Navarro are employed by and (with the exception of V. Bozon) own stock in Pfizer, Inc. Luis Camacho and Antoni Ribas receive honoraria and funding for research from Pfizer, Inc., and Antoni Ribas is also compensated for being an expert on the advisory board for Pfizer, Inc.

## Authors' contributions

JG-N, AG, and AR designed the study. LHC, B RS, JSE, JAG, and AR were responsible for patient care. BCA, YL, JJ, AA, and PdlR generated the immunologic data. BCA, VAB, CAB, ES, and AV collected these data. Data were analyzed by BCA, YL, VAB, CAB, JGN, and AR. The manuscript was written by BCA, JGN, and AR; and all co-authors reviewed the final manuscript.

## Consent

Written informed consent approved by the University of California Los Angeles and University of Texas MD Anderson institutional review boards was obtained from the patients for publication of this case report and any accompanying images. A copy of the written consent is available for review by the Editor-in-Chief of this journal.
